# KAT4IA: *K*-Means Assisted Training for Image Analysis of Field-Grown Plant Phenotypes

**DOI:** 10.34133/2021/9805489

**Published:** 2021-08-03

**Authors:** Xingche Guo, Yumou Qiu, Dan Nettleton, Cheng-Ting Yeh, Zihao Zheng, Stefan Hey, Patrick S. Schnable

**Affiliations:** ^1^Department of Statistics, Iowa State University, Iowa, USA; ^2^Plant Sciences Institute, Iowa State University, Iowa, USA; ^3^Department of Agronomy, Iowa State University, Iowa, USA

## Abstract

High-throughput phenotyping enables the efficient collection of plant trait data at scale. One example involves using imaging systems over key phases of a crop growing season. Although the resulting images provide rich data for statistical analyses of plant phenotypes, image processing for trait extraction is required as a prerequisite. Current methods for trait extraction are mainly based on supervised learning with human labeled data or semisupervised learning with a mixture of human labeled data and unsupervised data. Unfortunately, preparing a sufficiently large training data is both time and labor-intensive. We describe a self-supervised pipeline (KAT4IA) that uses *K*-means clustering on greenhouse images to construct training data for extracting and analyzing plant traits from an image-based field phenotyping system. The KAT4IA pipeline includes these main steps: self-supervised training set construction, plant segmentation from images of field-grown plants, automatic separation of target plants, calculation of plant traits, and functional curve fitting of the extracted traits. To deal with the challenge of separating target plants from noisy backgrounds in field images, we describe a novel approach using row-cuts and column-cuts on images segmented by transform domain neural network learning, which utilizes plant pixels identified from greenhouse images to train a segmentation model for field images. This approach is efficient and does not require human intervention. Our results show that KAT4IA is able to accurately extract plant pixels and estimate plant heights.

## 1. Introduction

**Figure 1:**
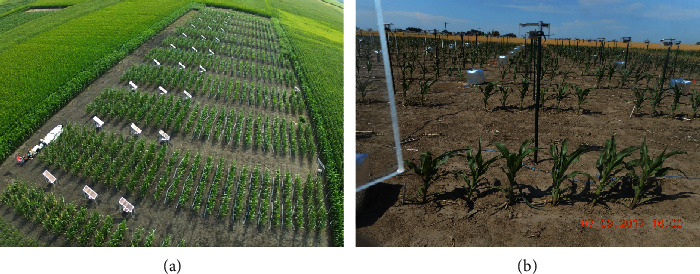
(a) An overview photo of the Iowa State field phenotyping system. (b) Raw RGB images of maize plants captured from the phenotyping facility.

One type of high-throughput phenotyping involves taking images of hundreds to thousands of plants simultaneously and continuously throughout their growth period. Substantial advancements have been made by engineers and plant scientists to enable the large-scale collection of plant images and sensor data in greenhouses and fields Chen´ e et al.´ [1]; Araus and Cairns [2]; Hairmansis et al. [3]; Fahlgren et al. [4]; Lin [5]; McCormick et al. [6]; Xiong et al. [7].  Figure 1 shows an example implemented by the Plant Science Institution (PSI) at Iowa State University, where cameras are placed in front of each row of plants in a field. These cameras are designed to take side-view photos every 15 minutes from 8 am to 5 pm each day. Side-view images provide access to different plant traits as compared to top-down images generated by gantry systems and UAVs (unmanned aerial vehicles). From the resulting images, we are able to process and extract phenotypic features such as plant height, width, and size and use those extracted features for subsequent genetic analyses. As compared to cameras mounted on mobile ground-based robots, using a system of this type allows simultaneous imaging of all plants, which offers advantages in understanding genetic variation in plant responses to varying environmental conditions.

Because high-throughput systems of this type can generate many images per day, image processing is generally required to extract numerical measurements of plant traits for downstream analyses [2, 8–11]. Plant object segmentation is the fundamental step in extracting phenotypic features from images [12, 13]. There are existing data analysis tools built for specific phenotyping systems, for example, Field Scanalyzer [14] by LemnaTec and CropSight [15], which uses Leaf-GP [16] for image processing. Those tools are all based on thresholding for image segmentation, which is accurate for greenhouse images, but less so for field images. Moreover, those tools are designed for top-view images and cannot be directly applied to side-view images. Image segmentation and trait extraction are still the current bottlenecks in many field phenotyping experiments. There are also systems, such as PlantEye by Phenospex, that generate and analyze 3D images obtained from above. However, 3D imaging technologies are expensive. Due to constraints, it is generally not possible to deploy hundreds of 3D lasers on large numbers of genotypes.

Separating plants from the background is much easier for greenhouse images where the background is homogeneous (usually white). Under such conditions, a thresholding algorithm can often provide satisfactory results [9, 12]. Thresholding is the simplest and the most commonly used method for image segmentation [10, 17]. Segmentation often involves classifying pixels using a cut-off value for pixel intensities. Thresholding can be applied on the average of red, green, and blue channels, on the green-contrast intensity [12], or on both [18].

**Figure 2:**
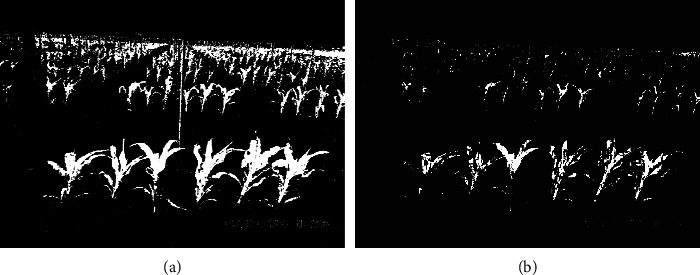
Thresholding segmentation method for Figure 1 using green-contrast intensity with weights $\left(-1/\sqrt{6},2/\sqrt{6},-1/\sqrt{6},\right)$, and threshold level 0.04 (a) and 0.08 (b).

However, thresholding methods do not perform well for field images, which typically have quite noisy backgrounds. As an example, the background in Figure 1 is a mixture of dirt and plant materials on the ground, poles, and silver heat shields that cover phenotyping equipment and plant shadows.  Figure 2 illustrates the performance of a thresholding method on ISU field images of maize, where a smaller thresholding value (0.04) maintains most parts of the plants but retains much of the background noise, while a larger thresholding value (0.08) removes most of the background noise but misses many plant pixels. Of particular concern, the ideal threshold for a given image is sensitive to the environment and time at which the image was taken. Hence, tuning thresholding values requires extensive human intervention and introduces an additional source of human bias.

A well-segmented plant image is key to accurate feature extraction, but traits such as plant height and width are particularly sensitive to background noise in images. To improve thresholding methods for greenhouse images, Adams et al. [8] made a thorough comparison for supervised learning methods trained on pixel intensities of plant RGB images acquired in a greenhouse, where the training data were obtained by unsupervised *K*-means clustering Johnson et al. [19]; Klukas et al. [20]. They demonstrated that neural network models are more accurate and robust at segmentation than traditional thresholding methods. For field imaging systems, there has been an increasing number of applications of convolutional neural networks (CNN) to plant phenotype extraction in recent years. Miao et al. [21] considered leaf counting of maize by a relatively shallow CNN; Lu et al. [22] employed deep CNN structures to count the number of tassels on field-grown maize plants; Aich et al. [23] used CNNs for estimating emergence and biomass of wheat plants. Other applications of CNNs on field images are described in Mohanty et al. [24]; Ubbens and Stavness [25]; Namin et al. [26]. U-net [27], which uses an autoencoder and decoder, is a recently developed popular CNN method for image segmentation. The idea of the U-net is to reconstruct an original image from its low-dimensional latent representation learned from the convolution of local structures of the training data. Despite the satisfactory performance of U-net on feature extraction, preparing the training data and annotating field images is still time and labor consuming because the field images are of high-resolution with noisy backgrounds.

To overcome the obstacle of preparing training data for field images, we provide the KAT4IA pipeline for plant feature extraction from field phenotyping systems based on a self-supervised learning algorithm for plant segmentation. The idea of self-supervised learning originates from semisupervised learning [28–30], which is a machine learning approach that combines a small amount of labeled data with a large amount of unlabeled data for training. Neural network-based semisupervised learning approaches can be found in [31, 32]. Semisupervised learning also has applications in plant phenotyping. For example, [33] considered a weakly supervised deep learning framework for sorghum head detection and counting, where the initial model is trained by a small dataset and is used to annotate new data. The annotation is then verified by human expert raters and fed back into the network to increase the size of training data. The proposed self-supervised learning approach generalizes semisupervised learning methods in the sense that no human labeled data are needed in the proposed approach. Self-supervised learning means our KAT4IA algorithm prepares the training data for in-field plant segmentation by itself without human labelling. This is possible for our problem because pixel intensities of greenhouse plants are similar to those of in-field plants, and greenhouse plant pixels can be easily obtained by unsupervised learning methods, like the *K*-means clustering algorithm. KAT4IA is able to automatically and robustly calculate plant traits from the ISU phenotyping system as shown in Figure 1 and to fit a nondecreasing functional curve for the extracted traits over the plant growth period. Compared to the method of Adams et al. [8] for greenhouse images, our pipeline has the following innovations: (i) extends the plant segmentation method to field images by transform domain learning; (ii) builds an automatic pipeline to separate the target plants and measure their traits; (iii) uses a nonparametric monotone fitting of plant traits that is free of model assumptions.

An important step in KAT4IA is to obtain an accurate segmentation of plants from field images. We construct a transform domain self-supervised neural network model, which uses plant pixels obtained by *K*-means clustering of pixels in greenhouse images, along with background pixels from field images to train segmentation models. This self-supervised method, which is novel in plant phenotypic analysis, can automatically and efficiently generate a large amount of supervised data by using plant pixels from greenhouse images and background pixels from field images as the training pixels. It is easy to implement and avoids expensive manual labelling for preparing training data. Postprocessing [13, 17, 34, 35] of the segmented image from the neural network model can be applied, such as median blur, erosion, and dilation operations. Using the segmented images, row-cut and column-cut algorithms in the pipeline were developed to separate the target plants by identifying the peaks of plant pixel proportions in image rows and columns. Plant features are then measured for each separated plant based on the segmented image. We also describe a refined feature extraction algorithm by pooling information of plant locations from a sequence of images taken over time in the same row of an experiment. In the last step, we fit a nonparametric and nondecreasing functional curve for the extracted plant trait. The advantages of nonparametric functional fitting over parametric modeling and point-wise analysis of variance for plant growth dynamics are discussed in Xu et al. [36]. Our method restricts the fitted curve to be nondecreasing which leads to a more accurate estimation for growth curve than the approach of Xu et al. [36]. Although we mainly focus on plant height measurement in this paper, our procedure can be easily extended to extract other plant traits such as size and width.

## 2. The KAT4IA Method

**Figure 3:**
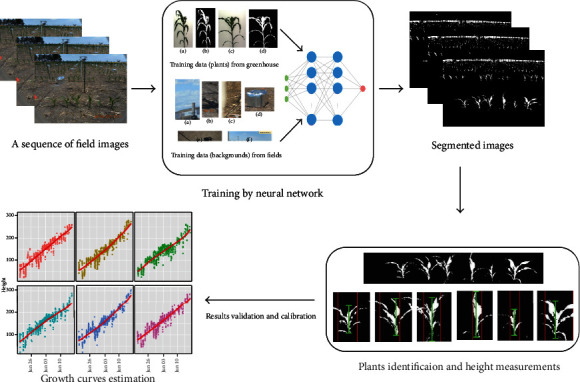
KAT4IA diagram. Subfigures from top left clockwise to bottom left illustrate the algorithm workflow from the original RGB images to the fitted growth curves.

The primary interest of this paper is to automatically extract the heights of all foreground plants in images recorded by cameras in the field (see Figure 1) and to use the heights obtained from sequences of photos to estimate plant growth curves. The workflow from the original RGB images to the fitted growth curve for each plant is summarized in Figure 3. The main steps are enumerated as follows. Detailed procedures for each step are explained in the subsequent subsections.

1. Construct the training data set for plant and background pixels, whereby the plant pixels are obtained using the *K*-means clustering algorithm applied on plant images from a greenhouse
2. Perform image segmentation using a neural network that classifies each pixel into 0 or 1 based on the RGB intensities of the training data, where 0 denotes background and 1 denotes plant
3. Identify plants of interest and measure their heights from the segmented images
4. Calculate the heights of plants from a sequence of images over the growing season
5. Estimate a plant growth curve using nonparametric regression with a nondecreasing mean function for each plant

## 3. Image Data

The image data used in this paper were taken from a rainfed (i.e., nonirrigated) field near Grant, Nebraska in 2017. One camera was installed for each row in two replications of 103 and 101 genotypes, respectively. Each row in each replication included up to six plants of a single genotype. Photos were taken at a frequency of 15 minutes, and the average number of photos taken by each camera was 1,719 and 1,650, respectively, for the two replications. We applied the KAT4IA pipeline to estimate growth curves for all the plant photos taken from the two replications. The raw field photos are high resolution (5152 × 3864) RGB images with intensity values of red, green, and blue channels between 0 and 255 for each pixel. We normalized the pixel intensities by dividing by 255, producing floating point numbers between 0 and 1. To increase computation efficiency, we also rescaled the image resolution to 1000 × 750.

## 4. Self-Supervised Learning

We considered self-supervised learning to classify each pixel of a field image into either a plant class or a background class. As preparing accurate training data is the most labor-intensive and time-consuming step in supervised learning, we deployed an efficient self-supervised learning method to automatically construct training data with labeled pixels for field images. To prepare training data for the background, it is straightforward to crop the image into pieces that only include the background. All the pixels in those pieces of images are labeled as background. For example, see the second panel in Figure 3, where the crops of background images include the dirt and plant material on the ground, sky, shadows, and the phenotyping equipment (e.g., the poles and silver heat shields).

**Figure 4:**
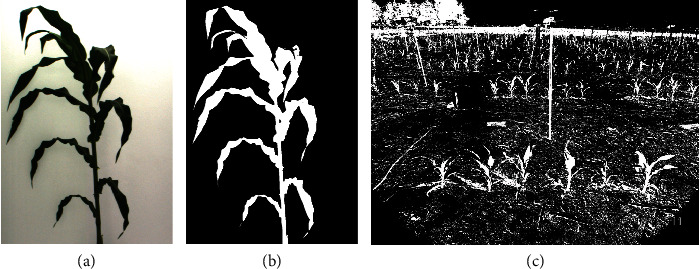
An example of training data (plant class) acquisition. Panel (a) is a cropped greenhouse images; panel (b) is the clustering result using the *K*-means algorithm (*K* = 3). The white parts are used subsequently as training data for the plant class. The number of clusters *K* could be chosen as 2. For *K* = 3, the third class gives the edge of the plant. Panel (c) presents the results of the *K*-means algorithm directly applied on a field image, which cannot separate the plant pixels.

To obtain training data for the plant class, however, it would be time-consuming to accurately crop the plant parts because of their irregular shapes and the noisy backgrounds in field images. Instead, we used plant pixels obtained from greenhouse images to train a model for field images. Specifically, we used images of plants that had been photographed in a well-controlled imaging chamber, where the backgrounds are much less noisy than field images. By cropping the greenhouse images, we obtained part of the plant in front of a background with a universal color; see panel (a) in Figure 4 as an example. This can be easily accomplished for greenhouse images. Because the cropped greenhouse images have only two distinct classes, the *K*-means clustering algorithm using a Euclidean distance metric can easily separate the plant pixels from the background pixels; see panel (b) in Figure 4 as the clustering result from the original image in panel (a). All the extracted plant pixels from *K*-means algorithm were collected as training samples of the plant class for field images. From panel (c) in Figure 4, we know that *K*-means clustering should not be applied on field images as it only works well for plant images with a universal background [8].

The key idea is to use the pixels from greenhouse plant images to train the pixel identifier for field images. Kernel density estimates of green contrast intensities for field background pixels, field-grown plant pixels, and greenhouse plant pixels are shown in Figure S1 in the supplementary material. From the figure, we see that although the green contrast density of greenhouse pixels is different from that of field-grown plant pixels, both densities deviate substantially from the distribution for field background pixels. The green contrast intensities for field-grown plant pixels tend to be much closer to the green contrast intensity distribution for greenhouse plant pixels than to the distribution for field background pixels. Thus, a classifier built on the greenhouse plant pixels and field background pixels is able to separate the field-grown plants from the background. Despite the changing lighting conditions in the field, our learning method produced good segmentation results under various field conditions and at different times of day, as demonstrated in the results section and the supplementary material section S4. Note that there is no need to have a perfect segmentation of the whole plant from the greenhouse, as we only need part of the plant pixels where separation from the background is easy and can be done by *K*-means clustering. Both the procedures to construct training data for the background and plant classes are easy to implement without human labelling and annotation. This makes supervised learning for plant segmentation possible at the pixel level.

Compared to traditional image segmentation like thresholding, our proposed method yields a more accurate results as indicated by Figure S2 in the supplementary material. Our proposed method is very efficient because we do not need the time-consuming and labor-expensive process of human labelling.

## 5. Segmentation by Neural Network

We used a training dataset generated as described above that consisted of 598,219 plant pixels from 6 greenhouse images and 2,728,415 background pixels in 19 cropped snippets from 6 field images of different environment conditions. For each pixel, we used its RGB intensities and those of the surrounding eight pixels (i.e., 3 × 3 pixels) as the input features. This results in 27 features for each pixel. Compared to neural networks with the target pixel only (i.e., no neighborhood), including the neighborhood information leads to a result with less background noise. The intuition is that plant and background pixels are more likely to be surrounded by pixels from their own category. In fact, the performance of neural networks with the target pixel only is more similar to the thresholding segmentation method shown in Figure 2. Compared to neural networks using 5 × 5 neighborhood pixels as input features, our 3 × 3 neural network has a similar segmentation performance and lower computation complexity. A more detailed comparison of neural networks with different neighbor sizes can be found in the supplementary material section S2.

A three-layer neural network under the API Keras in *R* was used to train the model. Specifically, the input layer had 27 nodes, and the first and second hidden layers had 1,024 and 512 neurons, respectively. The ReLU activation function was used between the input layer and the first hidden layer as well as between the first and second hidden layers. The output layer had one neuron which gives the predicted probability of a particular pixel belonging to the plant class. The sigmoid activation function is used between the second hidden layer and the output layer. The dropout rates at each hidden layer were chosen to be 0.45 and 0.35, respectively. The binary cross-entropy loss function with the Adam optimization algorithm (learning rate = 0.001) was used to evaluate the network. Finally, we used 20 epochs with batch size 1,024 to train the model. 1% of the training data were held out as a validation set before training.

**Figure 5:**
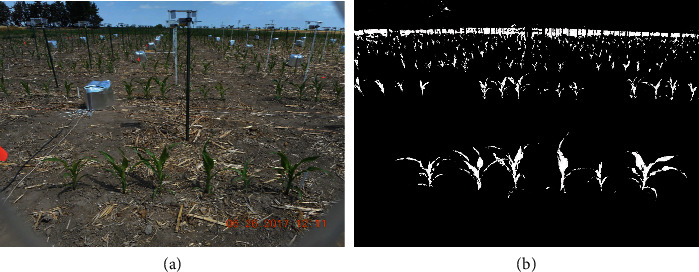
The original image (a) and segmentation result (b) from the self-supervised neural network model.

A cutoff threshold of 0.5 was used to classify the plant pixels, which means a pixel is classified as plant if its output probability from the neural net model is greater than 0.5. Our method is robust to this cut-off value. More discussion and results under different cut-off values can be found in the supplementary material section S3.  Figure 5 provides an example of the segmentation result by our neural network model. Most of the plants were precisely segmented with limited background noise. Even a corn field in the extreme background near the top of the image was correctly classified as plant. In contrast, the trees on the horizon were, for the most part, classified as background. More segmentation results for different plants and under various environmental conditions are shown in Figure S5 in the supplementary material. From those results, we can see that the proposed method is stable and robust under different weather and light conditions.

## 6. Plant Height Measurement from a Single Segmented Image

Based on the segmented images, we aimed to measure the height of the plants in the first (most forward) row of an image. As an example, there are six maize plants in the first row of Figure 5. This procedure constitutes identifying the first row by a row-cut algorithm and then separating each plant in the first row by a column-cutting algorithm before measuring the individual height of each plant.

### 6.1. Row-Cut Algorithm

**Figure 6:**
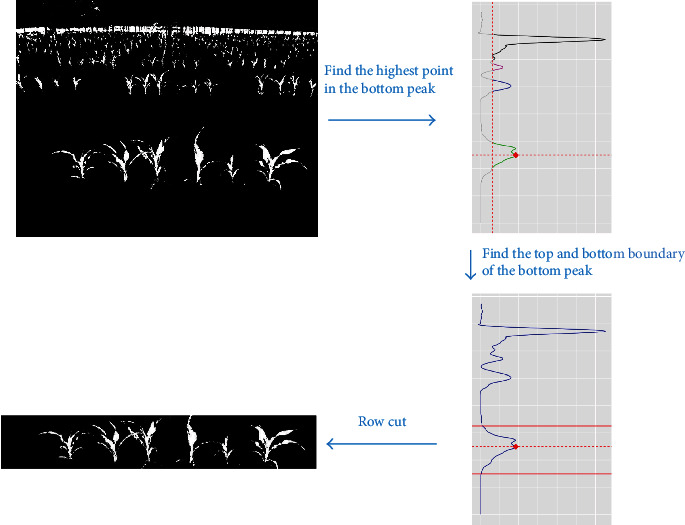
Diagram of the row-cut algorithm. Top left panel: the segmented image of plants from the neural network model; top right panel: the step of local maximum calling, which provides a separation of different peaks (illustrated by different colors) in the row mean curve and an identification of the maximum of the lower peak (denoted by the red point); bottom right panel: the step of peak region identification, providing the upper and lower boundaries of the bottom peak (denoted by the red solid lines); bottom left panel: the segmented and cropped first row of plants from the original image.

To separate the first row in an image, we use a row-cut algorithm which consists of local maximum calling and region identification. Specifically, row means are calculated for each pixel row of the segmented image, which gives the percentage of plant pixels in each row. Then, a local smoother (loess function in R) is used to smooth the row means. From Figure 6, we can see multiple peaks in the row mean curve, where the bottom peak corresponds to the front row of plants. To find the local maximum of the bottom peak, we threshold the row means by *R*_*v*_ = 10% percent of their global maximum value. This results in segments of row indices with values above the threshold, where two segments are considered to be separate if they are *S*_*r*_ = 10 pixel rows apart. The maximum of the bottom peak is the largest row mean in the first segment at the bottom of the image. See the illustration in the top right panel of Figure 6, where the red point denotes the maximum of the bottom peak (colored in green) identified by the procedure. Finally, to locate the region of the bottom peak, its upper and lower boundaries are chosen as the first pixel rows smaller than *R*_*u*_ = 7.5% and *R*_*l*_ = 2.5% percentage of its peak maximum when moving above and below from the center of the bottom peak. See the bottom two panels in Figure 6 as an illustration of this step. Our results show that this procedure can accurately separate the first row of plants and that it is robust to the tuning parameters *R*_*v*_, *R*_*u*_, *R*_*l*_, and *S*_*r*_ for all images analyzed. However, the appropriate values of those hyperparameters may vary in different experimental settings.

### 6.2. Column-Cut Algorithm

**Figure 7:**
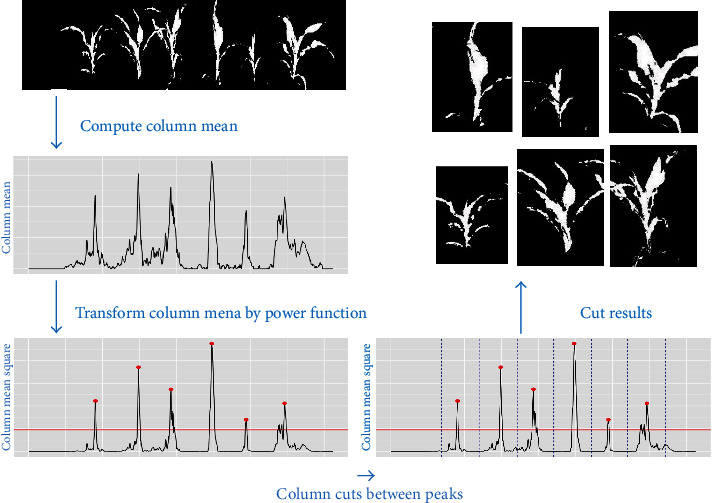
Diagram of the column-cut algorithm. Top left panel: the segmented first row of plants from the row-cut algorithm; middle left panel: the column mean curve; bottom left panel: the step of local maximum calling for the column mean curve, providing the maximum of each peak after the power transformation (denoted by red points); bottom right panel: the step of plant separation, where the cuts (blue dashed lines) between plants are calculated as the middle points of two adjacent peaks; top right panel: the segmented and cropped image for each plant.

Once the targeted row of plants is obtained, we separate each plant in that row using a column-cut algorithm. This algorithm is illustrated in Figure 7. Similar to the row-cut algorithm, the first step is to compute the pixel column mean values, which gives the column-wise percentage of segmented plant pixels. We applied a quadratic power transformation (i.e., *f*(*x*) = *x*^2^) to the column means, which magnifies the column peak maximal values so that it is easier to separate different peaks, as illustrated in the third step in Figure 7. Following the same strategy as the row-cut algorithm, we find the maximum for each peak by thresholding the squared column means at *C*_*h*_ = 20% percent of the overall maximum and obtaining segments defined by column indices with values larger than this threshold. Then, segments that are at least *S*_*c*_ = 50 pixel columns apart are considered to be from different peaks. The maximum value for each peak can be obtained as the largest squared column means in each segment. The cuts between plants are calculated as the midpoints between the indices of two adjacent peak maxima. Specifically, let {*I*_*p*_^(*j*)^}_*j*=1_^*m*^ be the indices of the column-mean peak maximum for the *m* plants. Let *I*_*c*_^(*j*)^, *j* = 2, ⋯, *m* be the indices of the cuts between plants. The left and right margin cuts are defined to be *I*_*c*_^(1)^ = max{*I*_*p*_^(1)^ − *D*_1_, 1} and *II*_*c*_^(*m* + 1)^ = min{*I*_*p*_^(*m*)^ − *D*_*I*_, *n*_*c*_}, respectively, where *D*_*I*_ = max_*j*∈{1, ⋯,*m* − 1}_[*I*_*p*_^(*j* + 1)^ − *I*_*p*_^(*j*)^/2] and *n*_*c*_ is the total number of columns.

### 6.3. Phenotype Measurements

**Figure 8:**
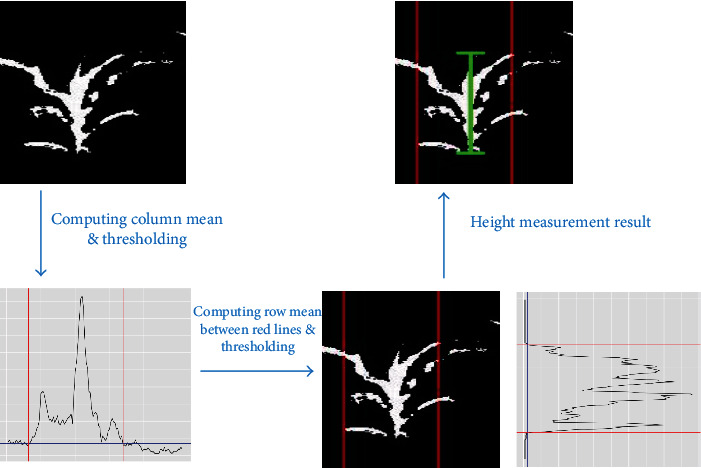
Diagram of the height measurement algorithm. Top left panel: the segmented image for a single plant from the row-cut and column-cut algorithms; bottom left panel: extracting the center part of the plant by thresholding (blue line) the column mean curve of the segmented image in the top left panel and identifying the left and right cuts (red lines); bottom right panel: the extracted center part (marked by two solid red lines) of the segmented image, and the height measurement by thresholding (blue line) the row mean curve of the center part of the segmented image; top right panel: the segmented image of a plant with the annotated height.

After making the row and column cuts, we can measure phenotypic traits for each plant. In this study, we focused on height measurement. The proposed procedure could, however, be easily adjusted to calculate plant width and size. For the height of each separated plant, we first computed the column means, then find the maximum value and the corresponding index of that maximum. Lastly, the left and right cuts were made to retain the center part of the plant: each cut was made at the pixel column closest to the column with the highest value among columns at which less than 10% of the maximum value was reached. The row mean values for the selected center part of the plant are computed, and the plant height is calculated as the index difference between the first row from below and the first row from above with mean values larger than 2.5% of the maximal row mean value. This procedure is illustrated in Figure 8.

## 7. Plant Height Measurement for Each Time Series of Images

In this section, we outline a refined height measurement procedure for a sequence of plant photos taken over time by borrowing information of plant locations across the time series of images. After conducting the above procedures for image segmentation, row cut, and column cuts, we can systematically study the growth trend of each separated plant over time and refine the column-cut algorithm that is based on a single image by considering a sequence of images from the same row, as the camera positions generally remain approximately fixed throughout the experiment. Consideration of a sequence of images can help to remove problematic images and images with overlapping rows of plants from which a clear separation of the plants in the front row is difficult.

**Figure 9:**
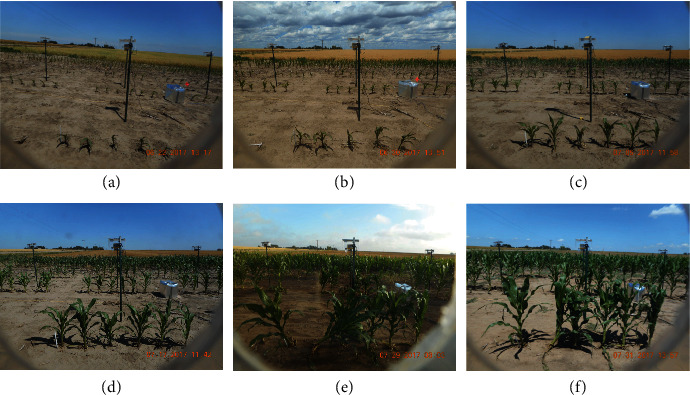
A sequence of field photos from a row of plants over the growth period.

Figure 9 shows a set of field photos of a row of plants taken by a single camera over time. Notice that the plant locations of plants are roughly the same across different photos. However, we cannot identify all six plants from every photo due to technical issues of the camera (panels (a) and (b) where the rightmost plant is obscured), strong wind (panel (e) where the second and third plants overlap) or the death of particular plants. Meanwhile, the row-cut algorithm requires a separation between the first (front) row and the second (background) row of plants, so that the bottom peak of the row means are separable from other peaks; see Figure 6. When the plants in the first row overlaps with the plants in the background, as shown in panel (f) of Figure 9, it is challenging to accurately measure plant height using computer vision methods. Our neural network algorithm is not able to separate the first row from the rest of the rows if they are overlapping in the perspective of the image. Hence, the current method is suitable for the earlier growth stages of field-grown plants. We explore potential solutions to this problem in the discussion.

**Figure 10:**
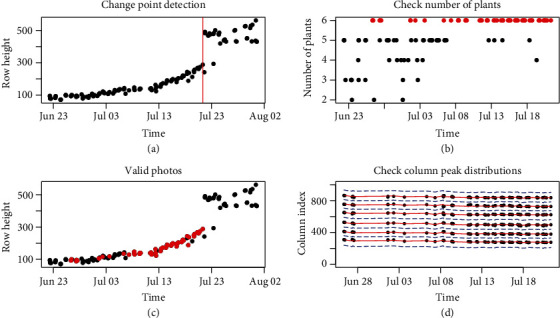
Refined height measurements for an exemplary sequence of images from one row. (a) Change point detection to identify the jump in the heights of the segmented rows, where the plants in the first row overlap with the background rows; (b) the number of identified plants in a given row over time; (c) the selected images (marked as red) for the growth curve analysis, which have 6 identified plants before row overlapping; (d) refining the column cuts for each image by pooling information of plant locations from other images in the same row over the growth period. The red solid lines are the estimated center of each plant over time, and the blue dashed lines are the refined column cuts.

To deal with the aforementioned challenges of the dynamic photos of plant growth, we have developed an algorithm to check image qualities to obtain more reliable estimates of plant height. This algorithm includes four steps as follows. First, the neural network segmentation model and the row-cut algorithm are applied to every photo in the sequence, and the heights of the segmented first row from each image are computed. We apply change point detection methods (via *changepoint* R package) to identify jumps in the heights of the segmented rows from the sequence of images. As illustrated in panel (a) of Figure 10, there is a clear jump in the row heights around July 21. This change point, denoted by the red vertical line, corresponds to the date when the front line of plants begins to overlap with the plants in the background, becoming inseparable. The current height measurement method only works for the early stages of plant growth when the target row of plants does not overlap with plants in the background. To separate plants from overlapped rows, we need to first obtain a good segmentation of all the plants that remove the background noise and then identify the targeted plants from the segmented image. The proposed method provides a solution to the first step of this process. We describe how to separate targeted plants when the rows are overlapping in the discussion section. We focus on measuring the plant heights of the front row prior to this change point. Second, the column cuts algorithm is implemented to count the number of plants in the front row for the segmented images from step one. The mode of these counts, denoted by *m*, is used as an estimate for the true number of plants in a given row over time. Because six seeds are planted in each row in this experiment, the modes for most of the rows are six during the growing season. We only consider those images with the number of plants in the first row equal to its mode *m*. This is illustrated in panels (b) and (c) of Figure 10, where *m* = 6 and the red points are the images with 6 identified plants over the time course. We compute the plant heights for those selected images for the time sequence of photos in the following steps.

**Figure 11:**
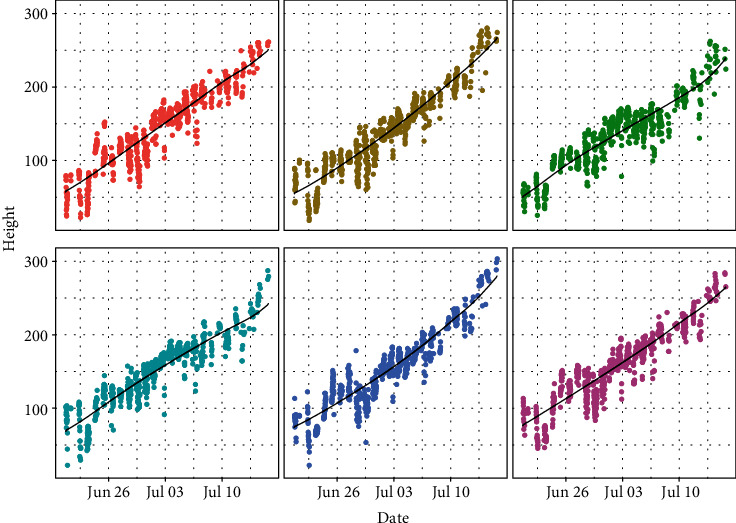
The fitted growth curves for each plant in a set of images from one camera. The points are the extracted plant heights from images, and the nondecreasing curves are the fitted values from the KAT4IA pipeline.

Given a row (camera), let *n* be the number of the selected images with *m* identified plants from the first two steps. In the third step, we refine the column cuts for each plant in a row by pooling information of plant locations from those selected *n* images. Let *I*_*p*_^(*i*, *j*)^ be the column peak index for the *j*th plant in the *i*th photo. The average column peak index for the *j*th plant can be computed as ${\bar{I}}_{p}^{\left(j\right)}=n^{-1}\sum_{i=1}^{n}\;I_{p}^{\left(i,j\right)}$. Note that the camera might slightly shift horizontally due to wind, which affects the position of the column peaks over time in a given row. However, the distance between two adjacent peaks should remain constant. Therefore, it is reasonable to stabilize the column peak index for the *j*th plant in the *i*th photo as ${\hat{I}}_{p}^{\left(i,j\right)}={\bar{I}}_{p}^{\left(j\right)}+\text{media}{\text{n}}_{j}\left(I_{p}^{\left(i,j\right)}\right)-\text{media}{\text{n}}_{j}\left({\bar{I}}_{p}^{\left(j\right)}\right)$, where the term $\text{media}{\text{n}}_{j}\left(I_{p}^{\left(i,j\right)}\right)-\text{media}{\text{n}}_{j}\left({\bar{I}}_{p}^{\left(j\right)}\right)$ adjusts the horizontal shift of the camera. The separation for each plant can be made at the average index of two adjacent peaks, as discussed in the “Column-cut algorithm” section. The red solid lines and blue dashed lines in panel (d) of Figure 10 show the stabilized column peaks and column cuts, respectively. Finally, we calculate the height of each separated plant as discussed in the previous section. The measured heights for the six plants in Figure 10 are shown in Figure 11.

## 8. Estimating Growth Curves

Plant heights are not expected to decrease during the growing season. Using the extracted heights from the plant images, we can fit a growth curve for each plant by nonparametric regression [37, 38]. However, the classical nonparametric curve fitting methods cannot ensure the nondecreasing property for the growth curve. To fit a nondecreasing function for the plant growth, following Dette et al. [39], we first apply a kernel-based estimation to fit an unconstrained growth curve $\hat{\mu }\left(t\right)$. Then, we construct a density estimate using the estimated values $\hat{\mu }\left(i/N\right)$ for *i* = 1, ⋯, *N*, where *N* is the total number of observations over time. It can be shown that integrating the density estimate from −∞ to *t* gives a consistent and nondecreasing estimator for *μ*^−1^(*t*) if *μ*(*t*) is a nondecreasing function. Thus, the estimator for *μ*(*t*) is also a nondecreasing function. To make the estimation more robust, outlying height measurements are detected based on the interquantile range of the residuals. Height measurements whose residuals are outside 3 times the interquartile range are ignored when fitting the nondecreasing growth curves a second time. The curves in Figure 11 are the fitted nondecreasing growth curves based on this method for six plants in one camera before the front row and the background rows overlap. Our method fit the data well with high *R*-square values. The goodness-of-fit results of the proposed method are reported in the supplementary material section S5.

## 9. Discussion

This paper describes a self-supervised method (*K*-means assisted training) to separate plants from background for field images and a computation pipeline to extract plant features (traits) from the segmented images. Our self-supervised learning approach is advantageous for high-throughput phenotypic analyses as no human labelling is required to construct supervisory training data. The absence of tedious human labelling makes up-scaling efficient and feasible. Our KAT4IA method is easy to implement and can be broadened to provide a variety of plant phenotypic analyses. Although this paper focuses on extracting height measurements, other features can also be extracted from the segmented images. For example, topological skeletonization can be applied to the postsegmentation binary images, and leaves can be separated based on skeleton-based computer vision methods.

The idea of transforming learning that uses greenhouse images to learn field images can be applied to various feature extraction problems. As many plant features, including height and number of leaves, have been extracted from greenhouse plant images [21], we can generate pseudofield images based on greenhouse images with their extracted plant features and build machine learning models on those pseudofield images to measure plant traits from field phenotyping projects.

As shown in Figure 10, the proposed method works for early stages of plant growth, during which the first row in the images does not overlap with plants in the background. Self-supervised learning methods can also be developed to separate the first row from the background plants if they overlap. This can be achieved in a two-step procedure. In the first step, the proposed segmentation method would be applied to segment all plants from the background. Training data of plant pixels from the first row and the background rows can be automatically formed from the images where the first row is separable. In the second step, using the training data, a convolutional neural network model can be constructed based on the pixel intensities from a small neighborhood of each pixel. In the same way, we have used greenhouse images to train self-supervised learning for field-grown plants, and we can use plant images in early growth stages to form self-supervisory information for the separation of plants in late growth stages.

The functional curve smoothing method is applied on each individual plant over time. Functional data analysis for genotype and treatment effects on plant growth can be conducted based on the fitted values from the nondecreasing functional curve. The “implant” package [18] can be applied on the smoothed plant traits for this purpose.

Currently, we do not have high-throughput field images with labeled plant pixels. In future work, results generated from our KAT4IA approach could be compared to results obtained by more labor-intensive approaches, such as using manually segmented images for supervised learning, obtaining manually measured heights of plants from images, or manually measuring plant heights in the field.

Finally, weeds were well controlled in our experiment, which can be seen from the original images. So, the proposed segmentation model does not consider weeds as the background. When weeds are prevalent, we could crop the part of the in-field images with weeds and use their pixels as part of the training data for the background class. A larger neighborhood size might be needed, as those surrounding pixels may be able to distinguish the structure differences between maize plants and weeds.

## Additional Points

*Additional Information.* The R code of the proposed pipeline, sample image data, and description are available on Github at https://github.com/xingcheg/Plant-Traits-Extraction.

## Supplementary Materials

Supplementary Materials The supplementary material includes the results of the proposed segmentation method under different environmental conditions, sensitivity analysis of the tuning parameters used in the proposed approach, and the goodness of fit evaluation of the fitted growth curves.
