# Supplementary material for: KAT4IA: K-Means Assisted Training for Image Analysis of Field-Grown Plant Phenotypes
Source: Plant Phenomics. 2021 Aug 3;2021:9805489. doi: 10.34133/2021/9805489 (PMC8358166; doi:10.34133/2021/9805489)
Supplement: Supplementary Materials — The supplementary material includes the results of the proposed segmentation method under different environmental conditions, sensitivity analysis of the tuning parameters used in the proposed approach, and the goodness of fit evaluation of the fitted growth curves. [file 9805489.f1.pdf]

Supplementary Material for "KAT4IA: K-Means Assisted Training for Image Analysis of Field-Grown Plant Phenotypes"

Xingche Guo[1], Yumou Qiu[1, *], Dan Nettleton[1], Cheng-Ting Yeh[2, 3], Zihao Zheng[3], Stefan Hey[3], and Patrick S. Schnable[2, 3, †]

[1]Department of Statistics, Iowa State University

[2]Plant Sciences Institute, Iowa State University

[3]Department of Agronomy, Iowa State University

[*]yumouqiu@iastate.edu

[†]schnable@iastate.edu

S1. Comparing the distributions of green contrast intensity between the greenhouse and field images

Kernel density estimates of green contrast intensities, i.e. $(2G-R-B)/\sqrt{6}$ distributions for the field background for field background pixels, field-grown plant pixels and greenhouse plant pixels are shown in Figure S1. From the figure, we see that although the green contrast density of greenhouse pixels is different from that of field-grown plant pixels, both densities deviate substantially from the distribution for field background pixels. The green contrast intensities for field-grown plant pixels tend to be much closer to the green contrast intensity distribution for greenhouse plant pixels than to the distribution for field background pixels. Thus, a classifier built on the greenhouse plant pixels and field background pixels is able to separate the field-grown plants from background.

S2. Comparing segmentation methods

We compare the thresholding segmentation method (using green-contrast intensity), K-means clustering of field-image pixel intensities, and our proposed neural network method for each of three neighborhood sizes. One example photo and its segmentation results can be found in Figure S2. Note that none of these methods requires expensive manual labelling for preparing training data. From Figure S2 (b) and (c), we can see that K-means with larger K will lead to a better result compared to small K; however, many background pixels are falsely classified as plant pixels by K-means. Our 1×1 neural network method is better than K-means; however, the 1×1 neural network method is visually more similar to the thresholding method

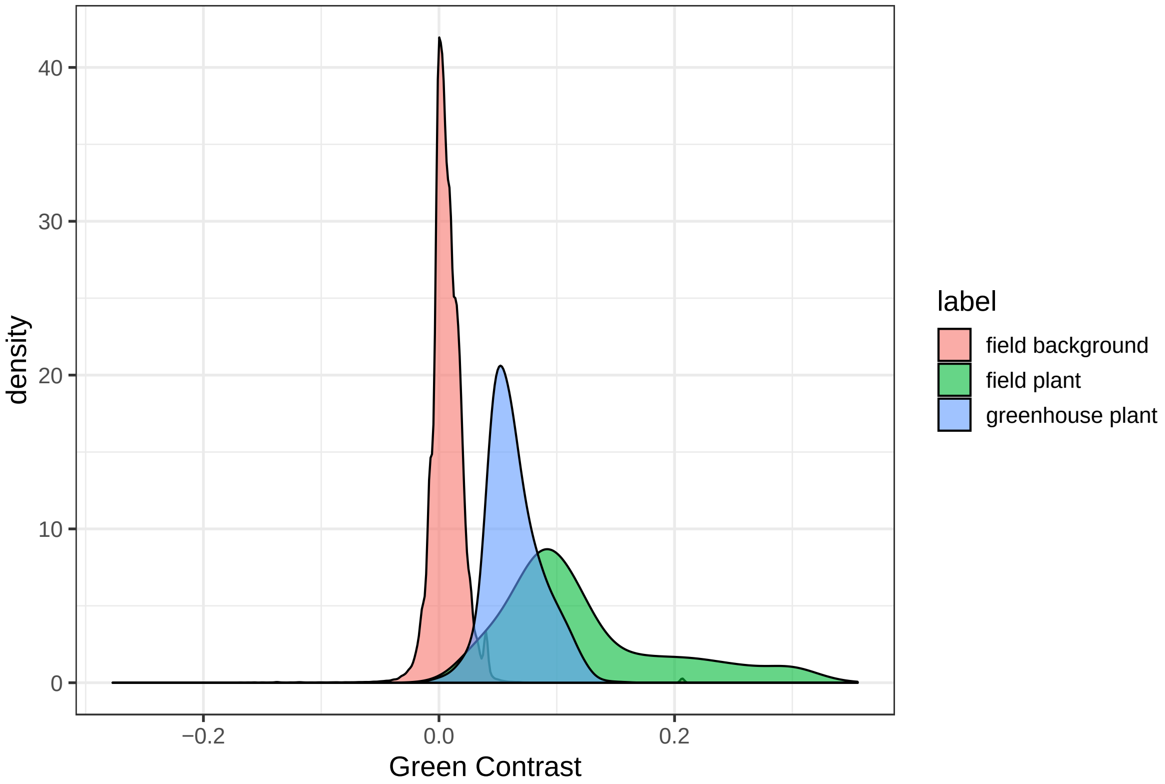

Figure S1. Distributions of green contrast intensity for greenhouse plant pixels, field plant pixels, and field background pixels.

(see (g)) rather than the neural networks that use 3×3 and 5×5 neighborhood information (see (e) and (f)). The results from the models with 3×3 and 5×5 neighborhoods are similar. As a smaller neighborhood size reduces the computation complexity of neural network approach, we choose the 3×3 neighborhood to construct our segmentation method. Figure S3 provides the comparison of the median height estimates of 23 randomly selected images from a photo sequence using our proposed algorithm. A local smoother is used to fit each growth curve. From Figure S3, we find that the neural networks with 3×3 and 5×5 neighborhoods perform similarly. The K-means algorithm fails to provide reasonable results. The neural network method using the target pixel only provides performance similarly to thresholding segmentation with threshold level 0.04. The thresholding segmentation method with threshold level 0.08 provides the smallest height.

S3. Comparing different cutoff threshold values of our proposed method

Figure S4 provides visual segmentation results and height measurements for three different cutoff threshold values (0.5, 0.9, and 0.95). Visually, the segmentation performs similarly across threshold values. When the cutoff threshold value is very high, say 0.95, there may be less noise, but the segmentation will provide almost the same height measurement result as for lower thresholds, which shows that our row-cut/column-cut algorithm is robust against noise.

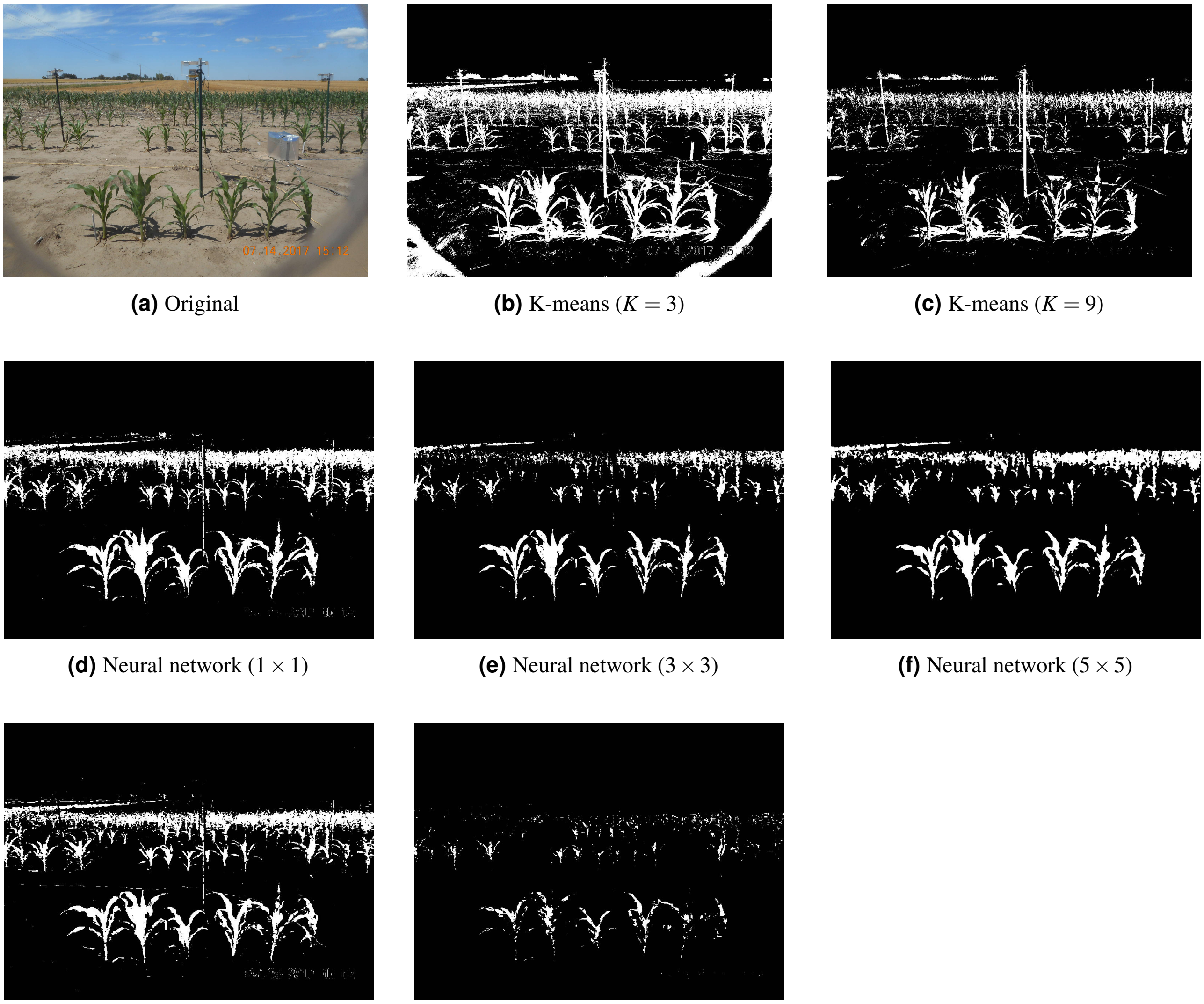

(a) Original **(b)** K-means ($K = 3$) **(c)** K-means ($K = 9$)

(d) Neural network (1×1) **(e)** Neural network (3×3) **(f)** Neural network (5×5)

(g) Green contrast thresholding (0.04) **(h)** Green contrast thresholding (0.08)

Figure S2. The segmentation results of the original image in panel (a) by K-means clustering with (b): $K = 3$ and (c): $K = 9$; the proposed neural networks with (d): the target pixel only, (e): its 3×3 neighborhoods and (f): 5×5 neighborhoods; and thresholding segmentation using green-contrast intensity with threshold level (g): 0.04 and (h): 0.08.

S4. Comparing segmentation results under different environmental conditions

Figure S5 provides segmentation results for two sequences of photos under five different environment conditions: dawn, sunrise, cloudy, foggy, and sunny, where the brightness and color scale of these images vary. From those results, we can see that our algorithm successfully segments most plant pixels without much background noise, which is sufficient to estimate the height accurately.

S5. Evaluating the goodness of fit of the fitted growth curves

To further illustrate that our proposed method leads to a stable estimation of growth curves, we evaluate goodness of fit by computing the R-squared values. The boxplots of the R-square values are presented in Figure S6, where panels (a) and (b)

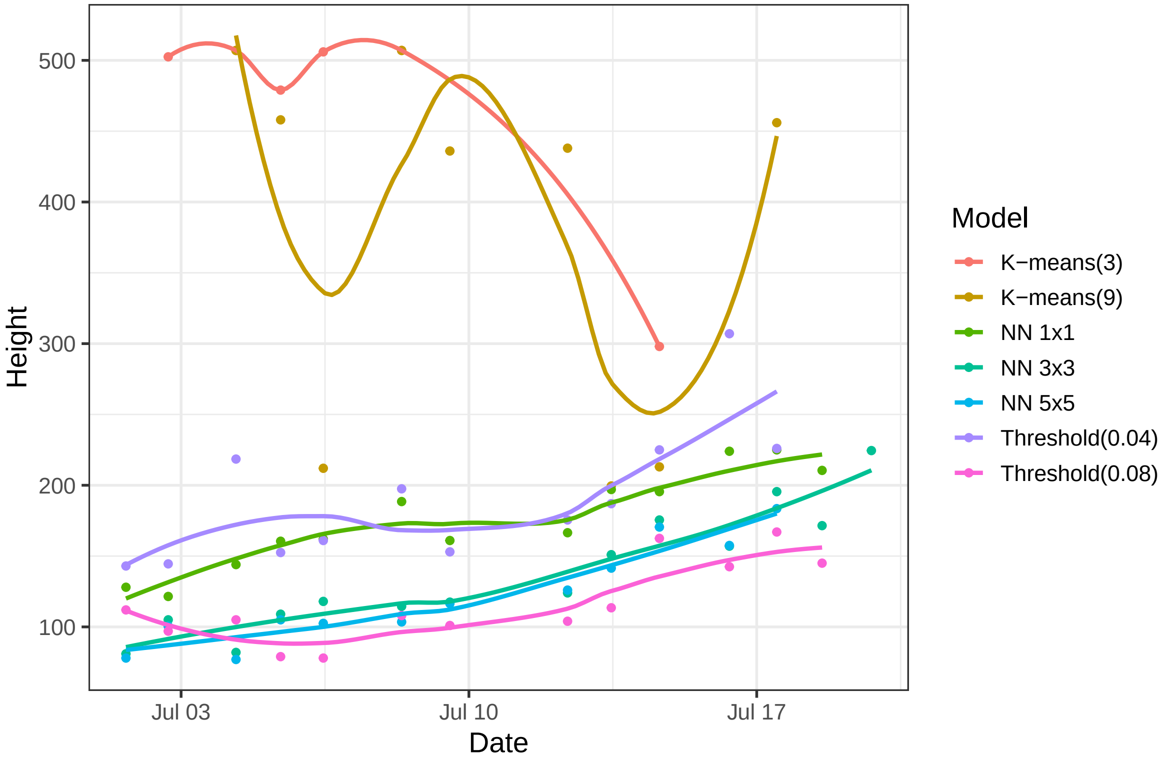

Figure S3. The comparison of the median height estimates of 23 random selected images from a photo sequence for the 8 different methods shown in Figure S2.

give the R-squared values for each camera (row) and for each plant position, respectively. We can see that our proposed non-decreasing nonparametric regression method can fit the height measurements well for most of the plants, given that most cameras have R-squared values around 0.8 or more.

The small R-square values for some plants from certain cameras as observed from Figure S6 in the supplementary material are due to plant death, overlapping of neighboring plants and changing weather conditions over the plant growth. Figure S7 provides the extracted plant heights and the fitted growth curves for a camera (camera No.45 in Figure S6) that shows a high spread of the R-square values among the six plants. Figure S8 provides the original images on four days from this camera with the vertical lines in four different colors indicating the heights measured by our KAT4IA pipeline. The measured heights of the six plants from the four images are also highlighted in Figure S7 with the same colors as those in Figure S8.

Figure S8 (a) shows one image of camera 45 taken at a time with little to no wind (on 07/11/2017), and panels (b) and (c) show two images of this camera taken under windy conditions (on 07/12/2017). From those figures, we can see that due to the wind effect, the measured heights for the middle four plants under the no wind condition are much higher than those under the windy condition, especially for the heights measured from panel (b) where the plants are severely bent left by wind. Meanwhile, the height of the right-most plant is affected by the leaves of the plant left to it as seen from panel (c). Panel (d) presents a case of imaging failure which causes under-estimation of plant heights. These reasons lead to a high variation of the extracted heights and low R-square values for some cameras.

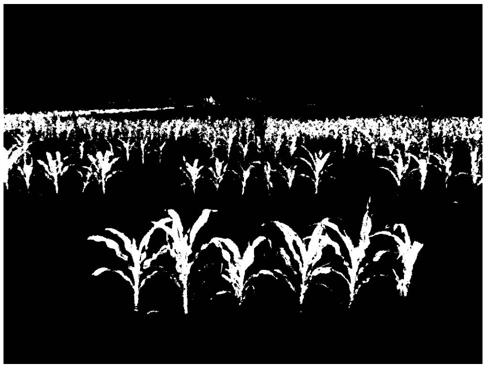

(a) threshold = 0.5

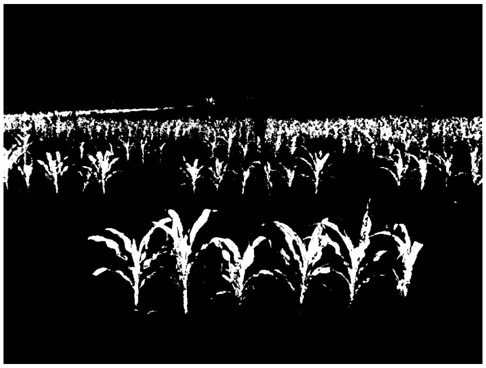

(b) threshold = 0.9

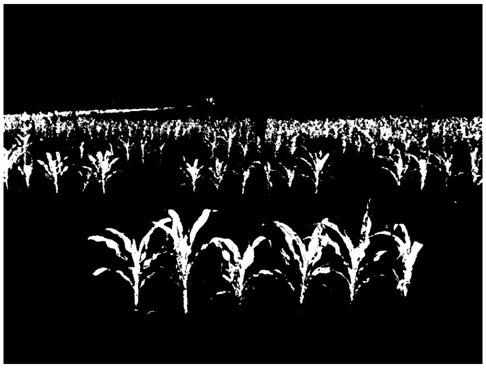

(c) threshold = 0.95

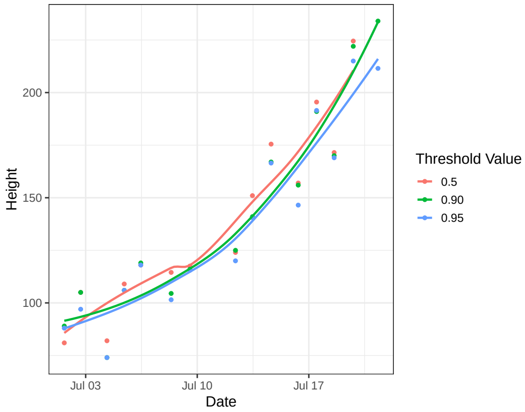

(d) compare height measurement

Figure S4. The segmented images using three cutoff threshold values are shown in (a) 0.5, (b) 0.9, (c) 0.95. The comparison of the median measured heights of 23 random selected images from a photo sequence is made among three different thresholds in (d).

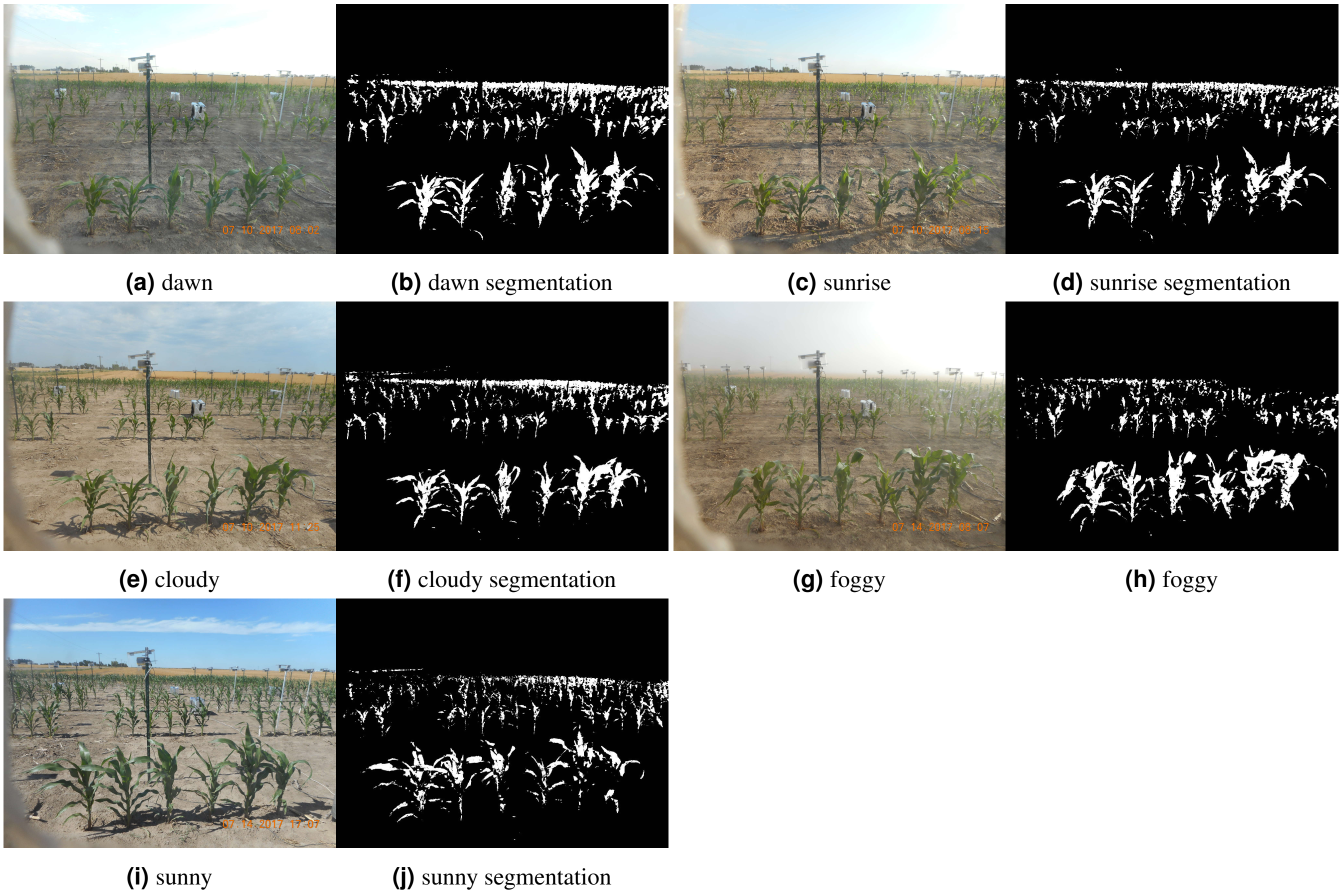

(a) dawn (b) dawn segmentation (c) sunrise (d) sunrise segmentation

(e) cloudy (f) cloudy segmentation (g) foggy (h) foggy

(i) sunny (j) sunny segmentation

Figure S5. Segmentation results of the proposed method for images under different enviromental and brightness conditions.

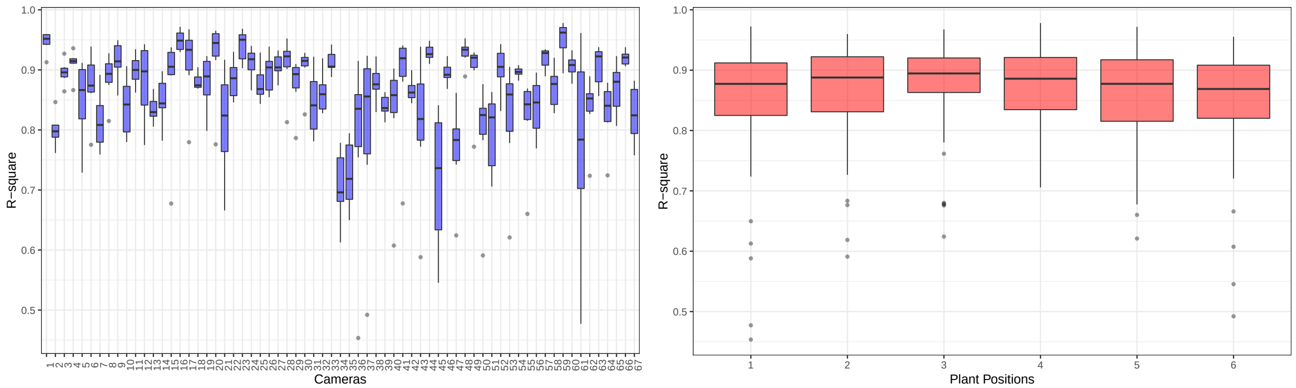

(a) (b)

Figure S6. The R-square value for 67 cameras × 6 plants. (a) the boxplot of R-squared values for each camera; (b) the boxplot of R-squared values for the 6 positions (i.e. left-most plants, second from the left plants, etc).

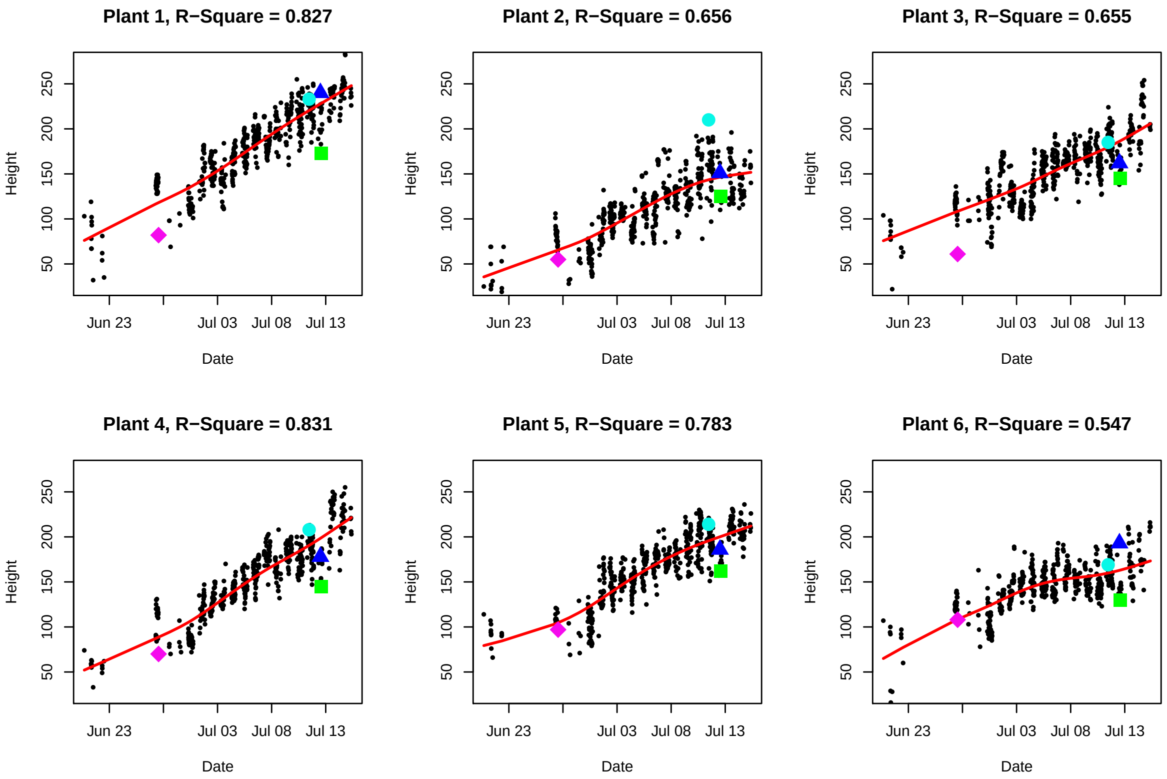

Figure S7. The extracted heights and the fitted growth curves by the proposed KAT4IA pipeline for each of the six plants in a set of images from camera No. 45. The highlighted points correspond to the cases shown in Figure R2.

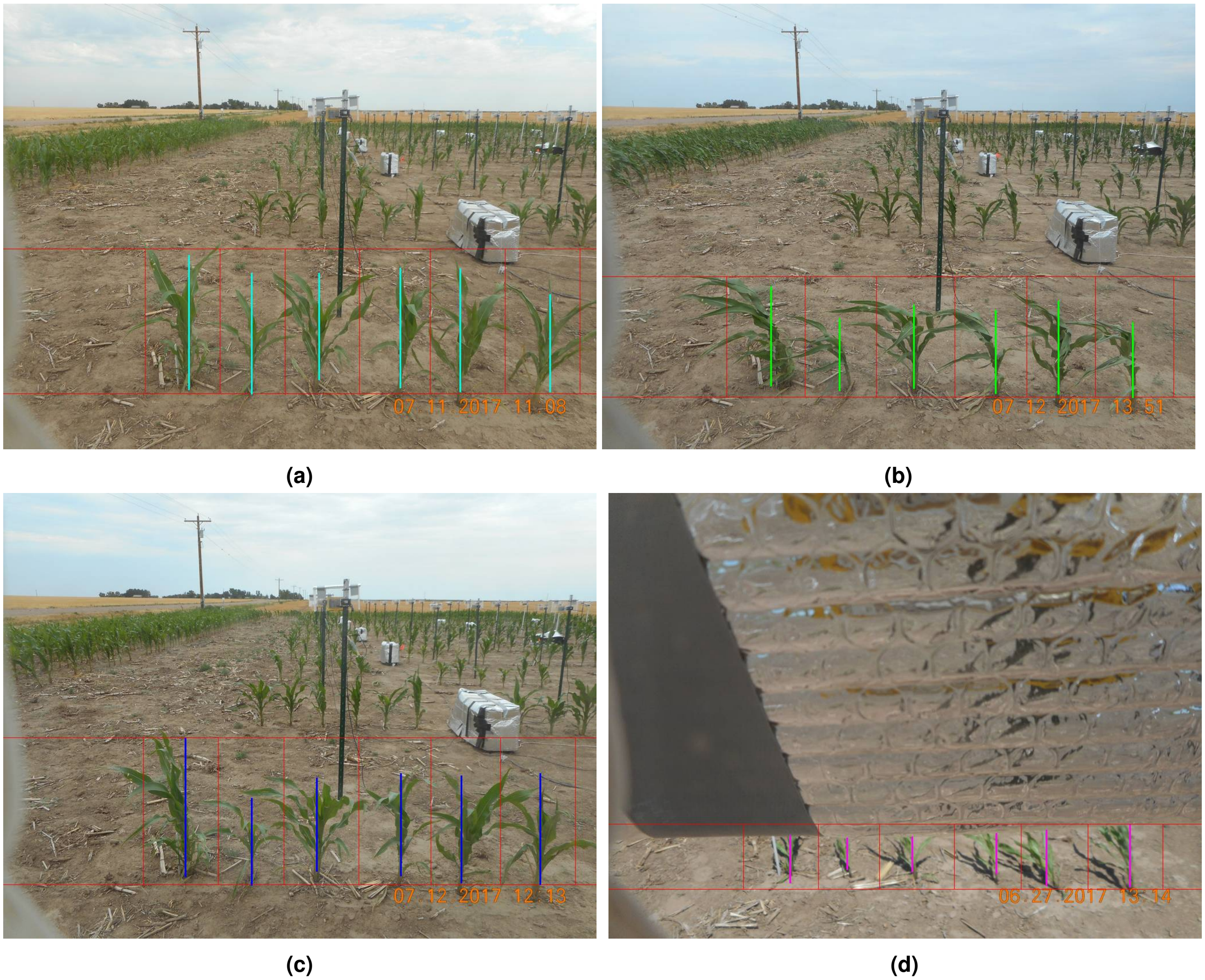

Figure S8. The original images from camera No. 45 under four different conditions. The red horizontal lines and red vertical lines correspond to the results from the proposed row-cut and column-cut algorithms. The vertical lines in four colors give visualization of the extracted heights by the proposed KAT4IA pipeline.
